# Prognostic factors for survival of women with unstable spinal bone metastases from breast cancer

**DOI:** 10.1186/s13014-015-0458-9

**Published:** 2015-07-15

**Authors:** Robert Foerster, Thomas Bruckner, Tilman Bostel, Ingmar Schlampp, Juergen Debus, Harald Rief

**Affiliations:** Department of Radiation Oncology, University Hospital Heidelberg, Im Neuenheimer Feld 400, 69120 Heidelberg, Germany; Department of Medical Biometry, University Hospital Heidelberg, Im Neuenheimer Feld 305, 69120 Heidelberg, Germany

**Keywords:** Bone metastases, Breast cancer, Radiotherapy, Survival, Stability

## Abstract

**Background:**

Bone metastases are an important clinical issue in women with breast cancer. Particularly, unstable spinal bone metastases (SBM) are a major cause of severe morbidity and reduced quality of life (QoL) due to frequent immobilization. Radiotherapy (RT) is the major treatment modality and is capable of promoting re-ossification and improving stability. Since local therapy response is excellent, survival of these patients with unstable SBM is of high clinical importance. We therefore conducted this analysis to assess survival and to determine prognostic factors for bone survival (BS) in women with breast cancer and unstable SBM.

**Methods:**

A total population of 92 women with unstable SBM from breast cancer who were treated with RT at our department between January 2000 and January 2012 was retrospectively investigated. We calculated overall survival (OS) and BS (time between first diagnosis of bone metastases until death) with the Kaplan-Meier method and assessed prognostic factors for BS with a Cox regression model.

**Results:**

Mean age at first diagnosis of breast cancer was 60.8 years ± SD 12.4 years. OS after 1, 2 and 5 years was 84.8, 66.3 and 50 %, respectively. BS after 1, 2 and 5 years was 62.0, 33.7 and 12 %, respectively. An age > 50 years (*p* < .001; HR 1.036 [CI 1.015–1.057]), the presence of a single bone metastasis (*p* = .002; HR 0.469 [CI 0.292–0.753]) and triple negative phenotype (*p* < .001; HR 1.068 [CI 0.933–1.125]) were identified as independent prognostic factors for BS.

**Conclusions:**

Our analysis demonstrated a short survival of women with breast cancer and unstable SBM. Age, presence of a solitary SBM and triple-negative phenotype correlated with survival. Our results may have an impact on therapeutic decisions in the future and offer a rationale for future prospective investigations.

## Background

Metastases in women with breast cancer most frequently occur in the skeleton [1]. Up to 2.6 % of all breast cancer patients already present with bone metastases at initial diagnosis and up to 15 % will develop bone metastases within 15 years [2, 3]. Particularly, metastases of the spinal column are a major cause of severe morbidity and reduced quality of life due to severe pain, pathological fractures, spinal cord compression and hypercalcemia [4, 5]. Additionally patients with unstable spinal bone metastases (SBM) are often immobilized or prescribed an orthopedic corset for the prevention of vertebral fractures and possible spinal cord compression. Treatment of SBM is multimodal including radiotherapy (RT), surgery and systemic treatments such as bisphosphonates [6]. Most commonly patients are treated with RT [7, 8] and a frequent treatment indication is instability [9]. In previous studies we were able to show that RT is capable of promoting re-ossification leading to increased stability of SBM. In those studies the analyzed patients showed only minor cancer-related morbidity during follow-up and reached comparably high survival rates. Additionally, we showed that the use of a validated scoring system to assess the stability of spinal bone metastases may prevent physicians from overdiagnosis of instability [10–12]. Since local therapy response is excellent, survival represents a major clinical interest in these patients with unstable SBM. Therefore, we conducted this analysis to assess survival and to determine prognostic factors for bone survival (BS) in women with unstable SBM from breast cancer.

## Methods

A total population of 92 patients with histologically diagnosed breast cancer and unstable SBM were treated with RT at the University Hospital Heidelberg between January 2000 and January 2012. All patients were included in this retrospective analysis. Inclusion criteria were an osteolytic phenotype, unstable vertebral body, location in the thoracic or lumbar spine and a minimum duration of follow-up treatment of six months. A total of 344 bone lesions in the thoracic and lumbar spine were identified in these patients. Bone metastases diagnoses were verified by computed tomography (CT). Many patients exhibited more than one treated lesion; only one lesion per vertebral body was included in the analysis. Bone metastases distal to the irradiated site were not included. The patients’ data were taken from the Heidelberg NCT Cancer Registry and are summarized in Table 1. Performance status was expressed using the Karnofsky Performance Status score (KPS) [13]. The specifications for an unstable vertebral body were tumor occupancy of more than 60 % of the vertebral body and pedicle destruction [14]. This study was approved by the Heidelberg Ethics Committee on 22 October 2012 (# S-513/2012).

**Table 1:** Patients’ characteristics

		Number	Percent
Age (mean, SD)		60.8 (±12.4)	
KPS	≤70	35	38.0
	>70	57	62.0
Localization	thoracic	67	72.8
	lumbar	25	27.2
Chemotherapy before RT	yes	53	57.6
	no	39	42.4
Radiological response at 3 months after RT	yes	29	31.5
	no	63	68.5
Radiological response at 6 months after RT	yes	42	45.7
	no	50	54.3
Bisphosphonates	yes	85	92.4
	no	7	7.6
Distant metastases	brain	6	6.5
	lung	13	14.1
	liver	19	20.6
	skin	2	2.2
Number of metastases	solitary	33	35.9
	multiple	59	64.1
Orthopedic corset	yes	33	35.9
	no	59	64.1
Pathological fracture before RT	yes	6	6.5
	no	86	93.5
Molecular phenotype	luminal A	55	59.8
	luminal B	8	8.7
	HER2	9	9.8
	triple negative	20	21.7

BS was defined as the time from initial diagnosis of SBM until death from any cause. The time of site irradiation was not equal to the time of initial diagnosis of bone metastases. Overall survival (OS) was defined as time from initial diagnosis of breast cancer until death from any cause. We estimated patients’ survival using the Kaplan-Meier method. Patients were censored on the basis of whether or not they were alive. Results were reported as the *p*-values of the log-rank tests. Multivariate analysis was performed to detect factors independently associated with BS using a Cox proportional hazards model. This regression analysis was performed by including the factors age (>50 years), Karnofsky Performance Status score (≤70 %), chemotherapy (ChT) prior to RT (no ChT), number of metastases (solitary metastasis), local response (response after 3 or 6 months), concomitant bisphosphonates (no bisphosphonates), orthopedic corset (no corset) and pathological fractures (no fracture). The results were reported as *p*-values, hazard ratios and 95 % confidence intervals (CI). For all analyses, a *p*-value of 0.05 or less was considered statistically significant. All statistical analyses were done using the SAS software version 9.3 (SAS Institute, Cary, NC, USA).

RT was carried out at the clinic of our department. After CT-assisted three-dimensional-simulation, RT was performed with 2-3 dorsal photon beams in the 6-MV energy range. The planning target volume (PTV) covered the specific vertebral body affected, as well as those immediately above and below it. The median individual dose in all patients was 3 Gy; the median total dose 30 Gy. The respective fraction and total doses were planned separately for each individual patient, depending on tumor histology, the patient’s general state of health, the current staging and respective prognosis.

## Results

**Fig. 1:**
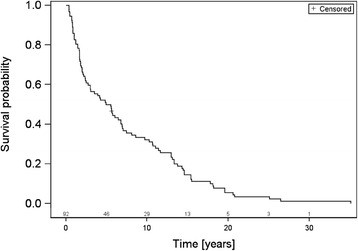
Overall survival

**Fig. 2:**
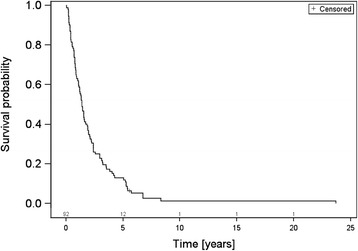
Bone survival

Mean age at first diagnosis of breast cancer was 60.8 years ± SD 12.4 years. OS rates after 1, 2 and 5 years were 84.8, 66.3 and 50.0 % respectively (Fig. 1). BS was 62.0 % after 1 year, 33.7 % after 2 years and 12.0 % after 5 years respectively (Fig. 2). Among the investigated possible prognostic factors only an age > 50 years (*p* < .001; HR 1.036 [CI 1.015–1.057]), the presence of a single bone metastasis (*p* = .002; HR 0.469 [CI 0.292–0.753]) and triple negative phenotype (*p* < .001; HR 1.068 [CI 0.933–1.125]) affected BS statistically significantly (Table 2). KPS, ChT prior to RT, local response, concomitant bisphosphonates, orthopedic corset and pathological fractures prior to RT did not statistically significantly influence BS (Table 2).

**Table 2:** Cox regression model of prognostic factors for bone survival

	HR	95 % CI	*p*-value
Age	1.036	1.015–1.057	<0.001
KPS	1.000	0.981–1.019	0.997
Chemotherapy	1.452	0.926–2.277	0.104
Number of metastases	0.469	0.292–0.753	0.002
Local response	1.071	0.661–1.736	0.779
Bisphosphonates	1.109	0.477–2.577	0.810
Orthopedic corset	0.821	0.500–1.350	0.437
Pathological fracture	1.533	0.614–3.827	0.360
Luminal A	1.538	0.998–2.167	0.945
Luminal B	1.152	0.876–1.825	0.745
HER2	1.472	0.977–1.957	0.628
Triple negative	1.068	0.933–1.125	<0.001

## Discussion

Breast cancer patients suffering from bone metastases of the spine represent a large patient group at most RT facilities. An important indication for RT treatment in these patients is instability, which is often associated with increased pain, profoundly reduced activity in daily life (ADL) and consequently severely impaired QoL. Unstable SBM may therefore be associated with shortened survival. We found OS and BS to be substantially shorter in our analysis with only 50 and 12 %, respectively, alive after 5 years compared to our previous study on osteolytic SBM in women with breast cancer [12]. Further studies have reported even worse survival rates in women with bone metastases [15], but this may be explained by a selection bias of only including patients with a follow-up of at least 6 months in our study. In an earlier small prospective study we already reported even lower survival rates in a population of patients with unstable metastases from various solid tumors [16]. In another study on patients with metastatic lung cancer we did not find any difference in survival between patients with stable and unstable SBM [17]. We believe that this was due to the extremely short survival time of those patients with metastatic lung cancer. Women with metastatic breast cancer have a more favorable prognosis than those with other solid tumors, e.g. lung cancer, and instability, possibly due to its associated morbidity, seems to be a relevant factor for long-term survival.

We found an age of more than 50 years, the presence of multiple SBM and triple negative phenotype to be associated with a worse prognosis after first diagnosis of bone metastases. In a recent study Bollen et al. [18] reported a median survival time of 22.5 months (95 % CI 18.0–26.9) for the receptor positive category and 6.7 months (95 % CI 2.4–10.9) for the triple negative category (*p* < 0.001). Therefore, patients with bone metastases from triple negative breast cancer have a significantly worse prognosis than those with a receptor positive phenotype.

According to the literature, another important prognostic factor for survival is the existence of additional extra-skeletal metastases [19]. In our analysis we were only able to demonstrate the prognostic relevance of age, the presence of a solitary metastasis and triple-negative phenotype. We believe this to be due to the small number of women with extra-skeletal metastases in our study cohort.

## Conclusions

This analysis demonstrated a short survival of breast cancer patients with unstable SBM. Importantly, we presented a correlation between age, presence of a solitary metastasis, triple-negative phenotype, and survival. This may have an impact on therapeutic decisions in the future. The results offer a rationale for future prospective investigations.

## References

[1] Lutz S, Berk L, Chang E, Chow E, Hahn C, Hoskin P (2011). Palliative radiotherapy for bone metastases: an ASTRO evidence-based guideline. Int J Radiat Oncol Biol Phys.

[2] Hagberg KW, Taylor A, Hernandez RK, Jick S (2013). Incidence of bone metastases in breast cancer patients in the United Kingdom: results of a multi-database linkage study using the general practice research database. Cancer Epidemiol.

[3] Berman AT, Thukral AD, Hwang WT, Solin LJ, Vapiwala N (2013). Incidence and patterns of distant metastases for patients with early-stage breast cancer after breast conservation treatment. Clin Breast Cancer.

[4] Janjan N, Lutz ST, Bedwinek JM, Hartsell WF, Ng A, Pieters RS (2009). Therapeutic guidelines for the treatment of bone metastasis: a report from the American College of Radiology Appropriateness Criteria Expert Panel on Radiation Oncology. J Palliat Med.

[5] Whyne CM, Hu SS, Lotz JC (2003). Biomechanically derived guideline equations for burst fracture risk prediction in the metastatically involved spine. J Spinal Disord Tech.

[6] Chow E, Zeng L, Salvo N, Dennis K, Tsao M, Lutz S (2012). Update on the systematic review of palliative radiotherapy trials for bone metastases. Clin Oncol (R Coll Radiol).

[7] Mitera G, Probyn L, Ford M, Donovan A, Rubenstein J, Finkelstein J (2011). Correlation of computed tomography imaging features with pain response in patients with spine metastases after radiation therapy. Int J Radiat Oncol Biol Phys.

[8] Wu JS, Monk G, Clark T, Robinson J, Eigl BJ, Hagen N (2006). Palliative radiotherapy improves pain and reduces functional interference in patients with painful bone metastases: a quality assurance study. Clin Oncol (R Coll Radiol).

[9] Souchon R, Feyer P, Thomssen C, Fehm T, Diel I, Nitz U (2010). Clinical Recommendations of DEGRO and AGO on Preferred Standard Palliative Radiotherapy of Bone and Cerebral Metastases, Metastatic Spinal Cord Compression, and Leptomeningeal Carcinomatosis in Breast Cancer. Breast Care (Basel).

[10] Foerster R, Habermehl D, Bruckner T, Bostel T, Schlampp I, Welzel T (2014). Spinal bone metastases in gynecologic malignancies: a retrospective analysis of stability, prognostic factors and survival. Radiat Oncol.

[11] Rief H, Bischof M, Bruckner T, Welzel T, Askoxylakis V, Rieken S (2013). The stability of osseous metastases of the spine in lung cancer—a retrospective analysis of 338 cases. Radiat Oncol.

[12] Schlampp I, Rieken S, Habermehl D, Bruckner T, Forster R, Debus J (2014). Stability of spinal bone metastases in breast cancer after radiotherapy: a retrospective analysis of 157 cases. Strahlenther Onkol.

[13] Karnofsky DA, Burchenal JH, MacLeod CM (1949). The Clinical Evaluation of Chemotherapeutic Agents in Cancer. Evaluation of Chemotherapeutic Agents.

[14] Taneichi H, Kaneda K, Takeda N, Abumi K, Satoh S (1997). Risk factors and probability of vertebral body collapse in metastases of the thoracic and lumbar spine. Spine (Phila Pa 1976).

[15] Oster G, Lamerato L, Glass AG, Richert-Boe KE, Lopez A, Chung K (2013). Natural history of skeletal-related events in patients with breast, lung, or prostate cancer and metastases to bone: a 15-year study in two large US health systems. Support Care Cancer.

[16] Rief H, Heinhold M, Bruckner T, Schlampp I, Forster R, Welzel T (2014). Quality of life, fatigue and local response of patients with unstable spinal bone metastases under radiation therapy—a prospective trial. Radiat Oncol.

[17] Rief H, Muley T, Bruckner T, Welzel T, Rieken S, Bischof M (2014). Survival and prognostic factors in non-small cell lung cancer patients with spinal bone metastases: a retrospective analysis of 303 patients. Strahlenther Onkol.

[18] Bollen L, Wibmer C, Wang M, van der Linden YM, Leithner A, Bunger CE (2015). Molecular phenotype is associated with survival in breast cancer patients with spinal bone metastases. Clin Exp Metastasis.

[19] Ahn SG, Lee HM, Cho SH, Lee SA, Hwang SH, Jeong J (2013). Prognostic factors for patients with bone-only metastasis in breast cancer. Yonsei Med J.

